# Using remote, spatial techniques to select a random household sample in a dispersed, semi-nomadic pastoral community: utility for a longitudinal health and demographic surveillance system

**DOI:** 10.1186/s12942-015-0026-4

**Published:** 2015-11-14

**Authors:** Amber L. Pearson, Amanda Rzotkiewicz, Adam Zwickle

**Affiliations:** Department of Geography, Michigan State University, East Lansing, MI 48823 USA; Department of Public Health, University of Otago, Wellington, New Zealand; Environmental Science and Policy Program, Michigan State University, East Lansing, MI 48823 USA; School of Criminal Justice, Michigan State University, East Lansing, MI 48823 USA

**Keywords:** Google earth, Random sample, Transect, Longitudinal household survey, Sampling frame

## Abstract

**Background:**

Obtaining a random household sample can be expensive and challenging. In a dispersed community of semi-nomadic households in rural Tanzania, this study aimed to test an alternative method utilizing freely available aerial imagery.

**Methods:**

We pinned every single-standing structure or *boma* (compound) in Naitolia, Tanzania using a ‘placemark’ in Google Earth Pro (version 7.1.2.2041). Next, a local expert assisted in removing misclassified placemarks. A random sample was then selected using a random number generator. The random sample points were mapped and used by survey enumerators to navigate.

**Results:**

We created a spatial sample frame and a random sample in 34.5 student working hours, 3 local expert hours and 1.5 academic working hours. Challenges included determining whether homes were occupied or abandoned, developing a protocol for placemark inclusion and quality issues with the aerial imagery itself. In the field, 175 sample points were visited and 170 of these (97 %) were actual households. The primary advantages of this method were the: (a) ability to generate a robust random sample in a rural and remote area; (b) lack of reliance on existing, external population data sources; and (c) relatively low levels of funding and time required.

**Conclusions:**

This method to develop a spatial sample frame was efficient and cost-effective when compared to in-field generation of a household inventory or GPS tracking of households. Utilizing a local expert to review the sample frame prior to field testing greatly increased accuracy. Overall, this method is a promising alternative to expensive and possibly biased household inventories or in-field GPS data collection for all households.

## Background

Obtaining a random sample for an in-person survey can be expensive and challenging, particularly in locations without readily-available census data. This process often involves systematic selection of a designated origin point (or individual) and sampling every *n*th house (or person) using known addresses (or a list of residents). Simple random sampling or systematic random sampling requires the use of census/administrative unit population information or household inventories, with households being randomly selected from the list [1].

In the absence of such information, other sampling methodologies must be employed. Alternatively, some researchers have reported the usefulness of transect sampling in the absence of address information, whereby one house is selected randomly and then transects are walked in random directions with sampling of houses encountered along each transect [2]. Other studies have used random point generators to select households [3].

When houses do not have addresses, houses are not arranged in a grid or not along a roadway, and houses are dispersed, these types of sampling become impractical or unrealistic. Rural, pastoral communities in sub-Saharan Africa, for example, are comprised of households that are often far apart, distant from roads, and not arranged in a purely linear fashion. Therefore, the probability of finding sufficient numbers of houses using a transect sampling methodology may be low. Another issue not typically addressed by conventional sampling methodologies, and relevant among pastoral households, is transience, even among ‘settled’ households [4]. Households may migrate in search of water and pasture in the dry season for a few months. However, many households may return to the same or nearby location when the rains return. As such, knowing the locations of homes in one or both seasons may be beneficial, particularly for longitudinal research projects.

One relatively novel and accessible method of establishing a robust sampling frame utilizes freely available aerial and satellite imagery to create a spatial sample frame, which can then be used to select a random sample. Specifically, Google Earth Pro images of the geographic study area can be used to identify and locate households, making an inexpensive sampling frame when household inventories or census data are unavailable. Random households can then be selected and easily found during subsequent surveying.

The identification of households for a sample frame using Google Earth has been employed in a few published studies, including in Iraq [5], Haiti [6] and Malawi [7]. However, in Iraq and Malawi, households were located in a denser, village-type setting and homes were arranged in a more linear fashion and therefore easily recognized in aerial imagery. In Iraq, Google Earth was used to select the first house to be sampled followed by on the ground random sampling from that household, within pre-identified population clusters. In Haiti, clusters were identified on Google Earth and then the locations of homes within those clusters were then captured. In all these studies using Google Earth, households were non-nomadic, making the identification of homes possibly simpler.

In this research, we tested the utility of Google Earth Pro to establish the household locations for the entire population in a dispersed community comprised of semi-nomadic households in rural Tanzania. The study area posed unique challenges and considerations including: (a) homes were composed primarily of earthen materials; (b) there were few roads present; (c) homes were primarily located in a non-linear fashion; and (d) several non-household structures may be located within the *boma* or compound. The aims of this research were to: (1) test a remote, spatial method of household identification to develop a sample frame; (2) and ultimately select a random sample to be later surveyed; (3) note the challenges, time spent, and potential uncertainties associated with this method for use in a longitudinal survey; and (4) report the in-field accuracy of this method.

## Methods

### Ethical approval

This methodology did not require ethical approval. The field-testing of the accuracy of the identified sample was deemed exempt by the Michigan State Institutional Review Board (# x15-423e).

### Study site

**Fig. 1:**
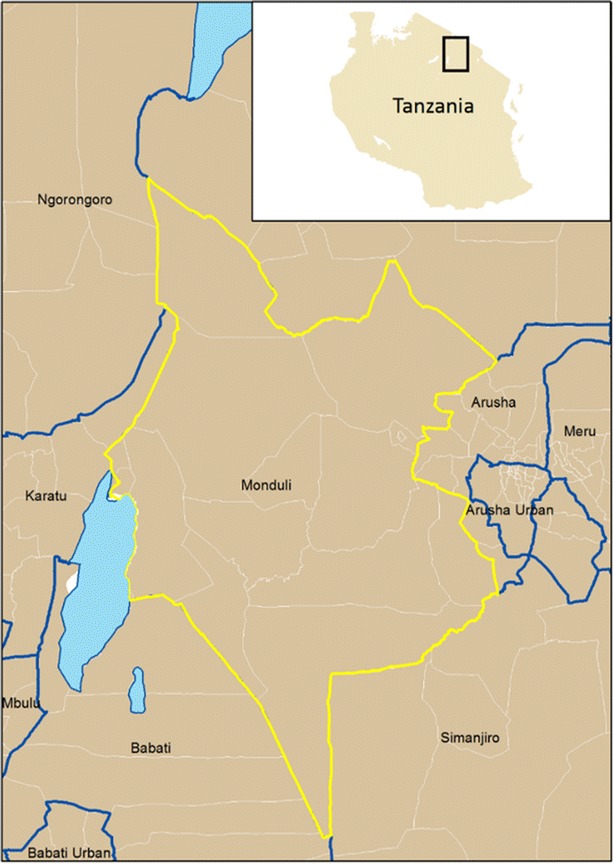
Monduli District (2012 census boundary), Tanzania

Naitolia (also spelled Nyatolia, area ~67 km^2^), in Monduli District, Tanzania (see Fig. 1), is a rural, agro-pastoralist village with a population of approximately 1800 people [8]. It receives varying rainfall, averaging to 650 mm annually [9]. The major ethnic groups are the Waarusha and the Maasai, speaking Maa and Swahili. Their families live in dispersed *bomas* or household compounds. *Bomas* usually consist of one or more home structure(s) made of earthen materials and a thatch roof, a kitchen structure and a kraal for livestock, which are encircled by thorny brush.

Historically, *bomas* were comprised of a number of Masaai households [10]. However, since the 1980s, *bomas* have progressively become smaller and now *bomas* are typically comprised of one household, particularly in Tanzania [11]. Most households are considered pastoralists in that they take cows to pasture and water, typically on foot or by donkey, and they depend on livestock production for their livelihoods, as source of food (both meat and milk) and a store of wealth. The most common source of income for approximately 70 % of households is from livestock sales [12].

The Naitolia community faces numerous challenges. Availability of water and secure access to quality water are major priorities for sustainable community development and public health promotion, for which recent interventions have been implemented by the Tanzania Partnership Project (TPP). To gauge the effectiveness of these interventions, Naitolia was selected as a study site for longitudinal health and demographic study.

### Identifying the geographic extent of the study area

The goal of our methodology research was to first locate every home in the study area (the sample frame) with the ultimate goal of using the sample frame to generate a random sample of households in Naitolia for inclusion in the longitudinal health and demographic study. The first step in achieving this goal was to delineate the spatial extent of the study area.

At the time of this study, the 2012 Population and Housing Census of Tanzania had not yet released village-level ArcGIS shapefiles on their website (or by email request). Therefore, the 2002 census boundaries for Naitolia village were used to define the spatial extent of the study area. The 2002 polygon shapefile for Naitolia was then exported as a.kml file, using ArcGIS v10.2 (Redlands, CA). The.kml file was then imported into Google Earth Pro v7.1.2.2041.

### Generation of the spatial sample frame

In order to achieve our ultimate goal of randomly selecting a sample of households within the study area, all households first had to be located to form the sample frame. To test the accessibility and potential for broad application of this method, the sampling frame was generated by a Master’s level graduate student with no prior experience using Google Earth Pro or ArcGIS. Working in Google Earth Pro, every single-standing structure or *boma* within the study bounds was located by placing an identifier or ‘placemark’. As the longitudinal survey to be conducted later will target households, and a *boma* is a familial compound consisting of numerous structures, *bomas* were treated as one point or household for the purposes of both the spatial sample frame and the survey. For each placemark, a short description of the features of the structure (e.g., large rectangle, small square, roofless structure, *boma*) were noted, along with the automated latitude and longitude coordinates of the placemark.

**Fig. 2:**
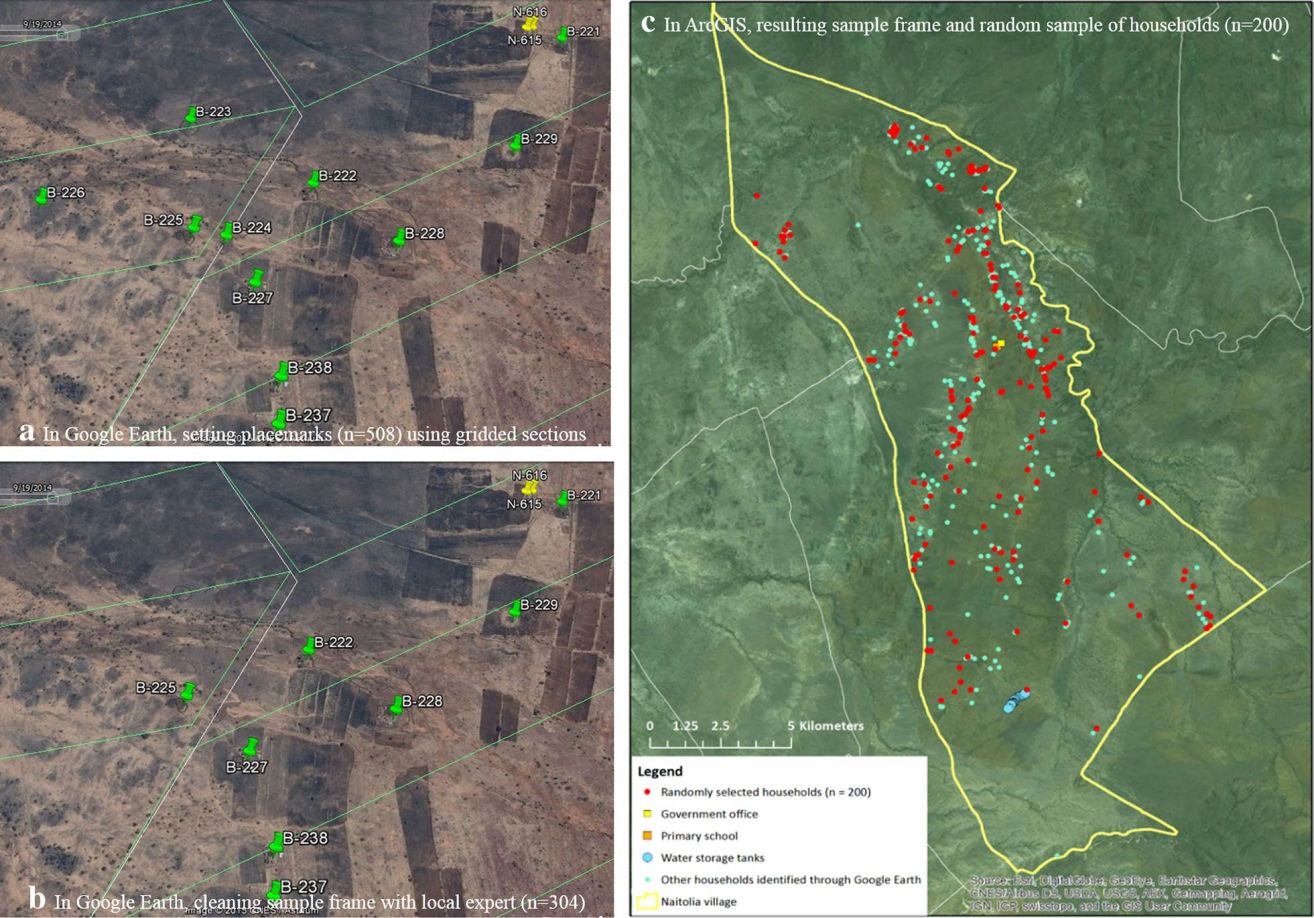
Methodological steps **a**–**c**

In order to systematically identify all households, the study area was divided into 71 horizontal sections (approximately 0.3–0.4 km wide) in Google Earth Pro, so that both upper and lower boundaries of the section were visible when viewed at an in-program ‘eye altitude’ between 4000–5000 feet. Within each section, the student scrolled from west to east, creating a uniquely numbered placemark for each identifiable single-standing structure or *boma* (see Fig. 2a). The two most recent aerial images available (January 2014/wet season and September 2014/dry season) were both examined, to minimize the influence of cloud cover and possible misinterpretation. The same section was then reevaluated, scrolling east to west, using a 2010/wet season aerial image, which had the highest resolution and best contrast of all available imagery. For example, trees and circular homes both appear brown in aerial images taken during the dry season, but stand in contrast in wet season images. Also, *bomas* are most clearly identifiable by their vegetative buffer, which is less distinguishable in the dry season. Notes were recorded for each placemark, including whether the structure was present across images from all years or not to accommodate for periodic dwellings common in a semi-nomadic society.

Ultimately, a total of 508 placemarks representing single-standing structures or *bomas* were generated, which took approximately 28.5 h to complete. In addition, metadata and attributes for each placemark were generated in Microsoft Excel, which took 3 h to complete, for a total of 31.5 h.

### Cleaning the spatial data frame using existing documentation and a local expert

In addition to homes, a primary school, water storage tanks, and a government office were also identified through existing TPP documentation. These structures were not included in the sample frame, but were included in the spatial dataset for the study area. Next, a local expert (Dr. Claude Mung’ong’o of University of Dar es Salaam) with experience in both aerial imagery and conducting research in Naitolia was recruited to assist in cleaning the spatial sample frame by removing placemarks which in his opinion did not represent households.

The expert reviewed the Google Earth Pro images containing the placemarks and eliminated 201 non-household placemarks (40 % of original placemarks), working alongside the student. Many structures were school-related buildings or surface water sources. These water sources were confused with tin-roofed structures, by the student, as they were highly reflective and rectangular. This process resulted in a final sample frame size of 307 households and took 3 h to complete (see Fig. 2b). For cartographic display purposes and for subsequent random sample selection, the.kmz file from Google Earth Pro containing the entire sample frame was imported and converted into a shapefile in ArcGIS (see Fig. 2c) which included the descriptive notes in the attributes table. Data were imported and checked by an experienced academic in 1 h.

### Generation of random sample

After performing a power calculation for the survey application, we determined the required sample size to be 200. To randomly select households (n = 200) from the spatial sample frame (n = 307), each point was assigned a random number ranging from 1 to 307, using Microsoft Excel. Then, using Stata v13 (College Station, TX), a set of 200 random numbers were selected without replacement. These selected points were then extracted from the sample frame to make a ‘sampled points’ shapefile and map to be used by survey enumerators to find the selected households during the longitudinal surveys (see Fig. 2c). This process was completed by an experienced academic and took 30 min.

### Challenges and potential uncertainties of this methodology

There were various challenges to the creation of the sample frame. Most notably, the most recent aerial image was taken during the dry season, which resulted in poor contrast between structures and the landscape. This was often remedied by referencing previous images to discern whether a structure was present. However, due to the nomadic nature of this population, it was possible for a structure to exist in one image and not another. Only structures present in the most recent image were given placemarks.

**Fig. 3:**
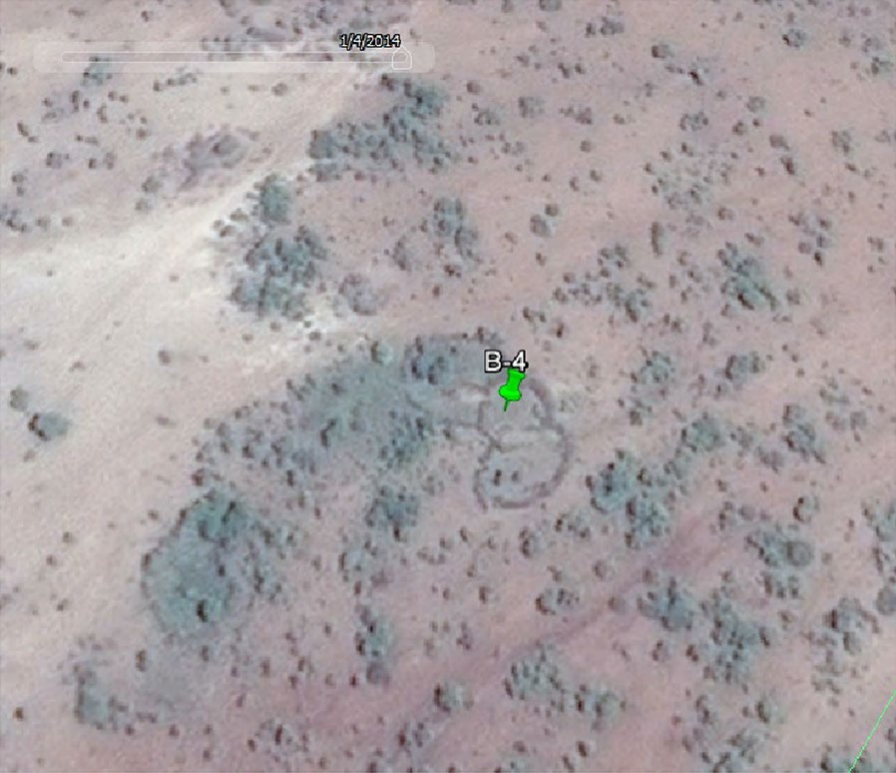
Presumably unoccupied *boma*

**Fig. 4:**
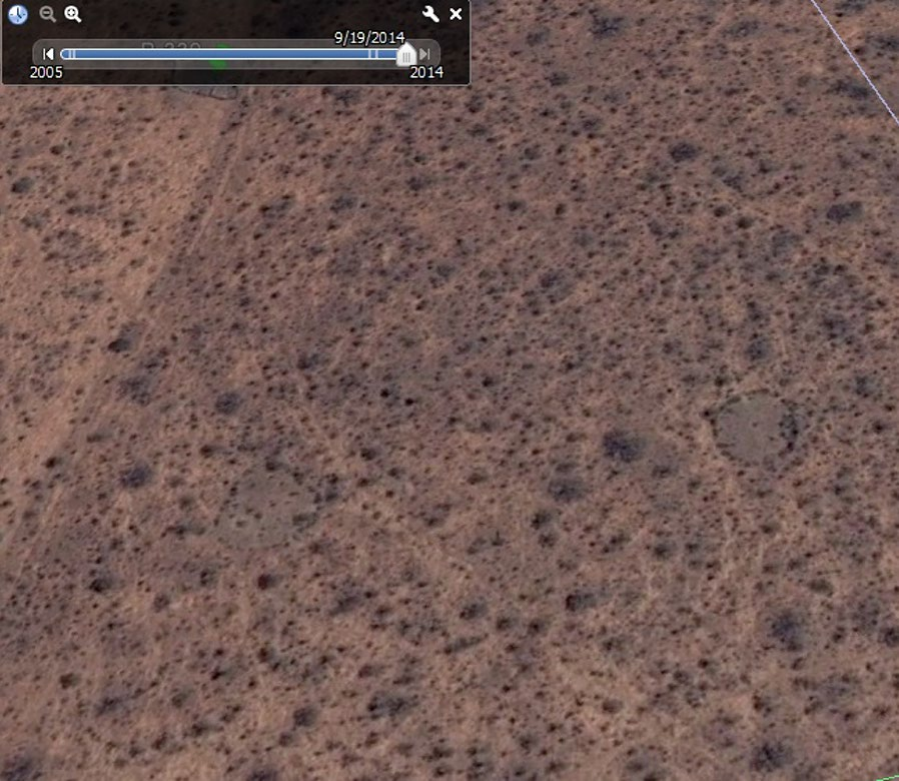
Visibly deteriorating *boma*

Also related to nomadism, existing but presumably unoccupied *bomas* (characterized by complete fences but lacking structures; see Figs. 3, 4 in the “Appendix”) were also given placemarks and their lack of occupancy noted in the metadata. Visibly deteriorating unoccupied *bomas* were not given placemarks. This step of the process, in particular, was the most time-consuming. A second complication was cloud cover. Fortunately clouds were a rare occurrence and could easily be avoided by viewing an older image. Additionally, a spatial mosaic discrepancy of up to 18 m was discovered between the two most recent Google Earth Pro images for some parts of northern Naitolia, as discovered by a misaligned road. If this image was used for setting the placemark, the spatial accuracy of the placemarks may have been affected and this was noted in the metadata. Cartographic examples of these issues are provided in the “Appendix”. Please refer to Table 1 for a summary of the challenges, advantages, uncertainties and time allotted for each step in the process, as well as the experience level of the user.

**Table 1:** Challenges, advantages, uncertainties and time allotted for each process involved in creation of sampling frame by student user with no prior experience

Process	Time required (h)	Challenges	Advantages	Uncertainties
Stage 1: Creation of placemarks for all single-standing structures and *bomas* (Google Earth Pro)	28.5	Poor visibility and or contrast	Previous images easily referenced	Potential for structures to be missed
		Time consuming	Easy to mark precise location (independent of occassional mosaicking)	Structures appear and disppear within short time frames so most recent image may not reflect current conditions
		Most recent image not available for all areas	No official data necessary to include all potential residences	
		Cloud cover		Potential for structures to be missed
		Bulky interface within Google Earth Pro when editing placemarks	Relatively quick process	Inaccuracy due to nomadism
Stage 2: Compilation of metadata (Microsoft Excel)	3	Have to enter each placemark individually	Easily imported to ArcGIS	Potential for data entry error
		Time consuming		
Stage 3: Cleaning sample frame with local expert	3	Orienting expert to this technique and aerial and satellite imagery	Elimination of non-household placemarks; robust sample frame	None

### In-field assessment of accuracy of this methodology

In the field, enumerators walked from house to house to enroll participants in the survey using tablets pre-loaded with aerial imagery of the study area and the locations of the selected homes to sample. Over a week, enumerators visited 175 of the 200 selected households, due to time constraints. Of these 175, only three were found to be something other than a house. One sample point was a guard house and two were man-made dams. In addition, two sample households were abandoned or the occupants had re-located the home. Overall, this method of generating sample points resulted in an accuracy level of 97 %, with 170 actual households out of 175 visited.

## Discussion

By using Google Earth Pro, aerial/satellite imagery and geographical methods, a non-expert user was able to create a spatial sample frame with attributes and metadata connected to each placemark in 31.5 h. This sample frame, which included many non-household structures, was substantially refined through the use of a local expert. After consultation with the expert and cleaning of the sample frame, we were then able to select a random sample for longitudinal survey application within 30 min by an expert geographer. Of the sample points visited, 97 % were actual households.

The primary advantages of this method were: (a) the ability to generate a robust and defensible survey sampling frame in a rural and developing area; (b) the lack of reliance on existing, external data sources such as household lists, small-area census data or detailed household maps; (c) the relatively low levels of funding and time required, and (d) the capacity to generate a sample frame prior to entering the field.

The ability to compare imagery from different years and seasons made it possible to better identify all homes and even those which are only occupied in the wet season, an issue which has made longitudinal analysis of semi-nomadic difficult in the past. From a longitudinal standpoint, the methodology outlined here provides the ability to return to the same household locations for future waves of prospective data collection during the wet season.

Using Google Earth Pro, a free and accessible software program, to develop a sample frame was efficient and cost-effective. However, there were challenges including cloud cover, mosaicking error, and the difficulty in discerning earthen structures in the dry season. Likewise, there were limitations to using an American graduate student who was unfamiliar with rural Tanzanian populations and landscapes. This was highlighted by the mis-classification of placemarks as households, an error discovered by a local expert. Therefore, it is highly recommended that the deployment of this method always include review by a team of local experts, rather than relying on one expert as done in this study. A further improvement on this study would be to include an accuracy assessment of a random selection of the structures which were identified as non-households by the local expert. The primary challenges to this method were developing a protocol for which structures to include (to minimize misclassification of houses) and issues with the aerial imagery itself (different resolution, mosaicking issues, cloud cover).

## Conclusion

Overall, this method of using freely available aerial and satellite imagery is a promising alternative to other expensive and time-consuming alternatives. Paired with help from a local expert, this method enabled us to draw a random sample of households in a rural, pastoralist community in Tanzania for recruitment into a longitudinal survey in 34.5 student working hours, 3 local expert hours and 1.5 academic working hours. This method may be usefully employed in a variety of settings: challenging environments with dispersed, rural communities comprised of earthen household structures and few roads and nomadic populations, as in this study, or possibly even dense city areas in developing countries with complex street arrangements and no formal addressing system such as urban slums.

## Figures and Tables

**Fig. 5:**
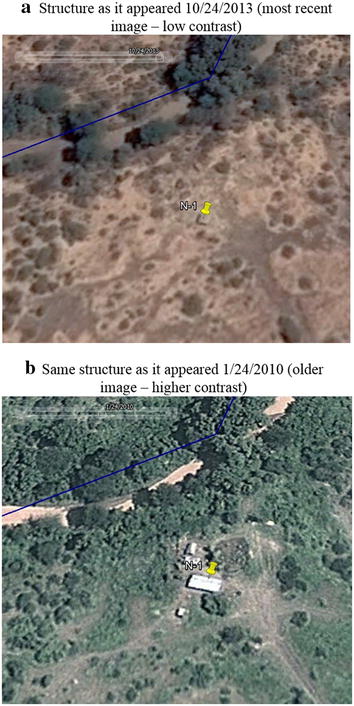
Challenge resulting from low image contrast and changes over time

**Fig. 6:**
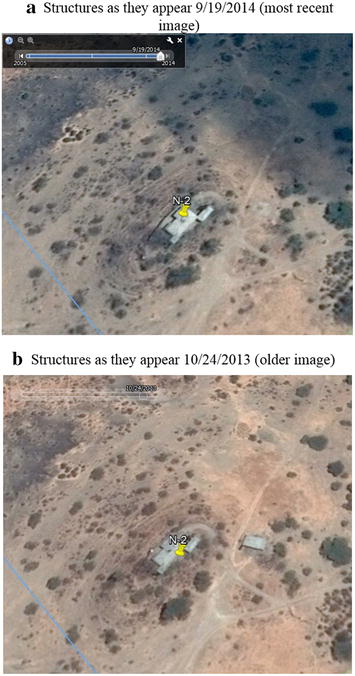
Appearance/disappearance of structure within two most recent images

**Fig. 7:**
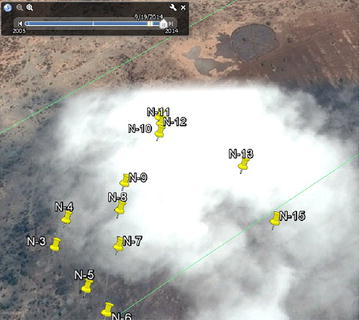
Cloud cover

**Fig. 8:**
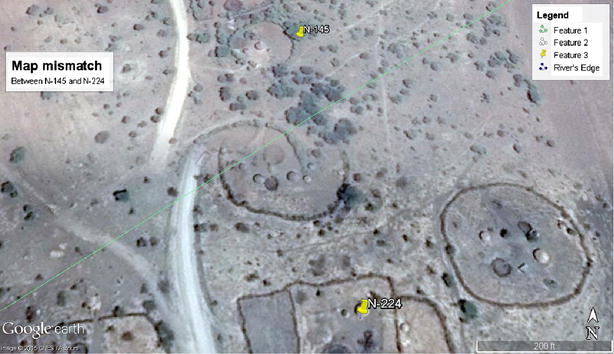
Evidence of mosaicking issue

## Appendix

See Figs. 3, 4, 5, 6, 7 and 8.

## References

[1] Kirkwood B, Sterne J (2003). Medical statistics.

[2] Morrison A, Astete H, Chapilliquen F, Ramirez-Prada G, Diaz G, Getis A, Gray K, Scott T (2004). Evaluation of a sampling methodology for rapid assessment of *Aedes aegypti* infestation levels in Iquitos, Peru. J Med Entomol.

[3] Kondo MC, Bream KD, Barg FK, Branas CC (2014). A random spatial sampling method in a rural developing nation. BMC Public Health.

[4] Pearson AL, Bradley DJ, Mayer JD (2015). Coping with household water scarcity in the savannah today: implications for health and climate change into the future. Earth Interact..

[5] Galway L, Bell N, Sae AS, Hagopian A, Burnham G, Flaxman A, Weiss WM, Rajaratnam J, Takaro TK (2012). A two-stage cluster sampling method using gridded population data, a GIS, and Google Earth(TM) imagery in a population-based mortality survey in Iraq. Int J Health Geogr.

[6] Wampler PJ, Rediske RR, Molla AR (2013). Using ArcMap, Google Earth, and Global Positioning Systems to select and locate random households in rural Haiti. Int J Health Geogr.

[7] Escamilla V, Emch M, Dandalo L, Miller W, Martinson F, Hoffman I (2014). Sampling at community level by using satellite imagery and geographical analysis. Bull World Health Organ.

[8] Census 2012, Village Statistics [http://www.nbs.go.tz].

[9] TANAPA (2001). Tarangire National Park General Management Plan/Environmental Impact Assessment.

[10] Bekure S, de Leeuw PN, Grandin B, Neate P (1991). Maasai herding: an analysis of the livestock production system of Maasai pastoralists in eastern Kajiado District, Kenya.

[11] Ikanda D, Packer C (2008). Ritual vs. retaliatory killing of African lions in the Mgorongoro Conservation Area. Tanzania. Endanger Species Res.

[12] University of Minnesota (2010). Whole Village Project Survey in Naitolia Village in Monduli District, Tanzania.

